# Does Free Route Implementation Influence Air Traffic Management System? Case Study in Poland

**DOI:** 10.3390/s21041422

**Published:** 2021-02-18

**Authors:** Ewa Dudek, Karolina Krzykowska-Piotrowska

**Affiliations:** Faculty of Transport, Warsaw University of Technology, ul. Koszykowa 75, 00-662 Warsaw, Poland; ewa.dudek@pw.edu.pl

**Keywords:** Air Traffic Management, free routing, air transport safety and quality, FMEA risk analysis

## Abstract

The issue addressed in this publication concerns new Air Traffic Management (ATM) functionality, identified in the Commission Implementing Regulation (EU) No 716/2014, known as Flexible Airspace Management and Free Route (FRA). The authors pose a question—does free route implementation influence air transport safety? What can be done to maintain the current level of safety and still implement modern solutions? To achieve the aim of this paper a developed concept of Risk Priority Number (*RPN*) calculation, with determination of main *RPN* components rating scales, in order to carry out the FMEA (Failure Mode and Effects Analysis) risk analysis of FRA implementation was done. The results allow lining up of the identified potential incompatibilities according to their criticality to the system. In effect it can be said that each modification in a management system, related to safety, influence the safety itself. Nevertheless, this influence does not always lead to negative impact.

## 1. Introduction

This article concerns issues related to quality and safety assurance in the broadly understood Air Traffic Management (ATM) system. Air transport serves people and organizations with its physical background (transport means, aerodromes and their equipment) as well as with modern technical solutions applied as interfaces between people and machines. In this process safety of human beings, their comfort and convenience as well as efficient system’s, functioning based on collected data are still top important in air traffic and flow management. The issues addressed in this publication concern new ATM functionalities identified in the Commission Implementing Regulation (EU) No 716/2014 [1] and deployed: to enable a more efficient use of airspace, to improve runway safety and throughput, to provide significant benefits linked to fuel consumption and delay reduction, to take care of air quality, etc.—to put it briefly: to better manage air traffic and its quality problems in a long term. Special attention is paid to one of the six ATM functionalities—Flexible Airspace Management and Free Route (FRA), known in Poland (selected case study location) as POLFRA and implemented by the Polish Air Navigation Services Agency (PANSA) in FIR (Flight Information Region) Warszawa on 28 February 2019. The paper directly addresses other publication [2], concerning ATM functionality development concept with particular analysis of free route airspace operations in Poland. It is an expansion of the analysis carried out till now, allowing further safety considerations and assurance. Some preliminary research has already been done. This time, however, the authors pose a question—does free route implementation (in Poland) influence air transport safety? How? To what extent? What else can be done not to deteriorate the level of safety available now, despite implementation of modern solutions? In order to give a reliable answer and in reference to the results obtained before, it was decided to develop and present in the following paragraphs, a concept of Risk Priority Number (*RPN*) calculation, with determination of the three main *RPN* components rating scales, in order to carry out the FMEA risk analysis of POLFRA implementation with a quantitative result showing the criticality of the identified potential incompatibilities as they have direct and indirect influence on ATM safety. This is the aim of this paper. Structure of the article, allowing the main aim’s achievement, is as follows: background of the research and literature review (chapter 1), Air Traffic Management—ATM with special highlight of free route airspace description (chapter 2), Failure Mode and Effects Analysis—FMEA of POLFRA implementation (case study), including Risk Priority Number—*RPN* calculation and its components determination (chapter 3) and last but not least—results and discussion (chapter 4) and conclusions (chapter 5).

### 1.1. Background of the Research

Air transport is a domain that used to develop dynamically within the last years (In this review Authors do not consider the influence of coronavirus pandemic on air transport development, as slowing down, noticed in this domain, is temporary. After this transitional period, according to latest forecasts, the dynamical development will continue and the problems addressed in this paper will again be actual). Actions resulting from this fact were focused on increasing the efficiency of air operations and airspace capacity, on improving runway safety and throughput as well as the quality and the timeliness of information sharing and on developing air infrastructure. This constant and undeniable development had many advantages from the passengers’ point of view, but at the same time enforced on Air Traffic Management (ATM) services and aerodrome’s managers elaboration and implementation of new measures to assure the adequate and efficient use of airspace capacity, efficient use of collected data and what is even more important the adequate level of air traffic’s safety with real time mitigation of incompatibilities. Moreover, the solutions already deployed must have been controlled in accordance with the principle of continuous improvement and proactive approach to safety management. Due to epidemiological situation all over the world air transport growth has suddenly been stopped. Nevertheless, the rules in force so far must be obeyed and implemented for smaller air traffic also. Furthermore, Eurocontrol forecasts (based on [3]) show that in 2021 traffic situation is foreseen to be at the level of 73% of flight numbers in comparison to year 2019, while in 2022 it should regain 89% and in 2023 even 96% of the operations performed in 2019. Which means that the observed development slowdown is temporary and the problems addressed in this paper will again be actual soon.

**Figure 1:**
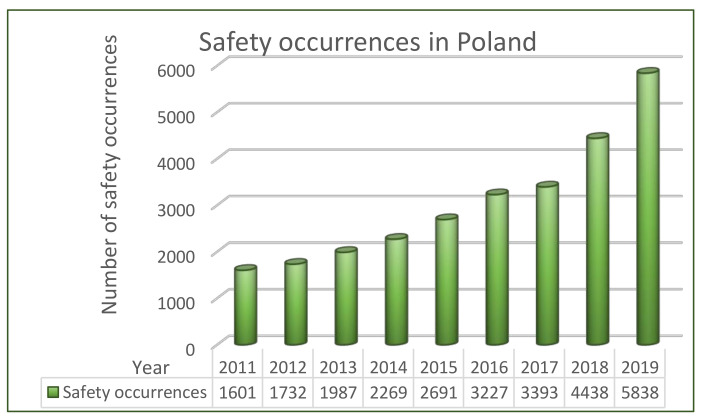
Number of safety occurrences in Poland, years 2011-2019, own work based on [4].

Within the last few years, an increase in the number of air safety occurrences can be noticed, see Figure 1, based on [4].

The reasons thereof may be numerous. First of all, the growing number of air operations performed as well as attended passengers, which generally means bigger traffic, may cause the increasing number of aviation occurrences. On the other hand, it may be an effect of higher reporting culture. Irrespectively of the reasons, the main focus must be placed on ensuring that the level of air traffic and transport safety on the macro scale, irrespective of the current development, is not deteriorated. In practice, two strategies of continuous process’ improvement must be applied: principle DMAIC (Define-Measure-Analyse-Improve-Control) and PDCA (Plan-Do-Check-Act). Both require that safety analysis are performed in a cyclic way and repeated periodically considering changes over time. This explains the research done to perform the risk analysis, described in the following part of the article. Moreover, the analyzed problem is a practical one, it’s a challenge that was faced daily by aerodromes’ managers, air carriers, ground handling, etc. as safety of air operations has a direct impact on human/passenger life).

### 1.2. Analysis of the Current Situation in Legal Requirements and Literature

Air transport is a discipline very heavily entrenched by legal regulations. Due to its international and transborder character those regulations must be considered respectively on global, European and national level. For the issue under consideration—air traffic safety, ATM, quality assurance, safety management, risk analysis and free routing—as main international documents ICAO Annexes to the Convention on International Civil Aviation with the accompanying Manuals should be recognized, in particular ICAO Annex 19 [5]. On European level Eurocontrol specifications as well as numerous Commission Regulations must be taken into account. As background documents, concerning ATM modernization, the Single European Sky (SES) general idea and Single European Sky Air Traffic Research and Development (SESAR) project, presented in various documents, should also be listed. Eurocontrol even developed a reporting framework harmonized with EU and ICAO regulations, the Toolkit for ATM Occurrence Investigation (TOKAI), which allows a structured and unified reporting for Air Navigation Service Providers (ANSPs). It was presented in [6]. On national level Polish National Civil Aviation Safety Program [7] and its accompanying documents released by the Civil Aviation Authority of the Republic of Poland or Polish Air Navigation Services Agency such as [8] should be taken into account.

The issue of safety management and risk analysis was a subject of other authors’ publications e.g., [9,10,11]. Risk assessment methods are also being addressed in other numerous papers, e.g., [12,13,14]. The issue of events probability estimation may be found in [15] or [16] for example and their consequences in [17]. Fortunately, scientists also noticed a possibility to forecast and assess the consequences of aviation safety occurrences, e.g., in [18]. Other techniques are also present in [19] or [20]. A subject matter expert review for a temporal topic modelling applied to aviation safety reports showed Robinson in [21]. A new approach to Air Traffic Management safety in contrast to the traditional one is presented by various authors, both in Poland—in e.g., [22], where safety is considered to be treated as a constrain instead of being the main goal, as well as in Europe—e.g., [23], in which a new look at global safety monitoring is addressed. Free Route implementation initial analysis may be found in [2], for example. Beyond that benefits and challenges of its deployment at a large scale in European airspace are being studied in [24] for example. As a continuation in [25] the application of Free Route Airspace in Northern Europe with reference to Functional Airspace Blocks (FAB) is described, expanding the analysis on two SESAR project solutions. Although it is the European region in which the freedom of route selection is so strongly developed, publications from other parts of the world can also be found. In [26] the case of Manila FIR is being considered, taking into account future growth of air traffic volume in the Philippines. Adequate simulations are done and a comparison of free route approach and traditional currently used structures is placed, showing environmental benefits as well as capacity and ATC workload improvement. Besides, perception of safety culture in China was a matter of research in [27].

The conclusion that can be drawn from the review made is that similar challenges of free route implementation are to be faced not only in Poland (FIR Warszawa) or even Europe but in other countries or regions also. In reference to the FRA research problem it is worth noticing that generally it is not widely discussed in the literature. In particular, there is a lack of publications concentrating on safety approach and FRA risk assessment. That is why, in the following paragraphs its deployment is analyzed from the safety point of view.

## 2. Air Traffic Management and Free Route Airspace

### 2.1. Air Traffic Management

The idea of Air Traffic Management—ATM (according to [28]) understood as dynamic, integrated management of air traffic and airspace—safely, economically and efficiently—through the provision of facilities and seamless services in collaboration with all parties and involving airborne and ground-based functions) modernization with specified functionalities to be improved, goals to be reached and deadlines to be meet appears in [1]. The mentioned document identifies the following areas to be improved within the coming years (the last ones till January 2025):

1. Extended Arrival Management and Performance Based Navigation in the high density Terminal Maneuvering Areas with two sub-functionalities:
  - arrival management extended to en-route airspace,
  - enhanced terminal airspace using RNP-based operations,
2. Airport Integration and Throughput with five sub-functionalities:
  - departure management synchronized with pre-departure sequencing,
  - departure management integrating surface management constraints,
  - time-based separation for final approach,
  - automated assistance to controller for surface movement planning and routing,
  - airport safety nets,
3. Flexible Airspace Management and Free Route with two sub-functionalities:
  - airspace management and advanced flexible use of airspace,
  - free route,
4. Network Collaborative Management with four sub-functionalities:
  - enhanced short term ATFCM (Air Traffic Flow and Capacity Management) measures,
  - collaborative NOP (Network Operations Portal),
  - calculated take-off time to target times for ATFCM purposes,
  - automated support for Traffic Complexity Assessment,
5. Initial System Wide Information Management (SWIM) with six sub-functionalities:
  - common infrastructure components,
  - SWIM technical infrastructure and profiles,
  - aeronautical information exchange,
  - meteorological information exchange,
  - cooperative network information exchange,
  - flight information exchange,
6. Initial Trajectory Information Sharing with no sub-functions defined.

In this paper functionality No 3 - Flexible Airspace Management and Free Route is being analyzed. It was selected as the first one to be worked on due to the closest deployment target date, which was set on 1 January 2022 for Free Route operations.

### 2.2. Free Route Airspace—Introduction, Benefits, Challenges

**Figure 2:**
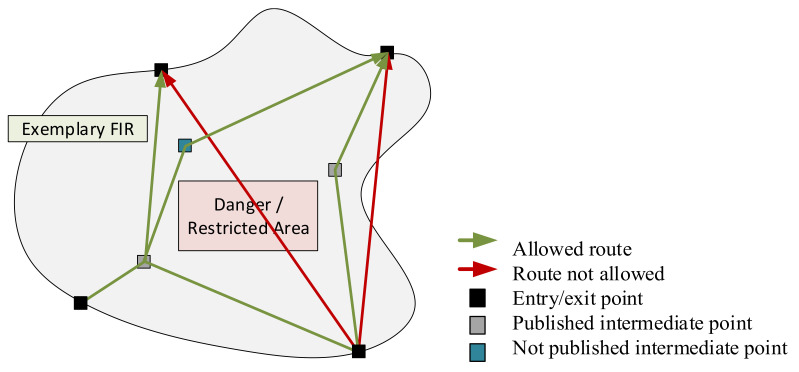
Exemplary FIR with allowed and not allowed routes indication (adapted from [30].

Free Route Airspace (FRA) according to its definition gained from [29] is a specified airspace within which users may freely plan a route between a defined entry point and a defined exit point, with the possibility to route via intermediate (published) waypoints, without reference to the ATS (Air Traffic Service) route network, subject to airspace availability (see Figure 2). Within this airspace, flights remain subject to air traffic control.

Subject to airspace availability, the green routes should be accepted, while the red ones—rejected by Air Traffic Control, due to danger/restricted area crossing or route indication out of FRA/FIR boundaries. Instead of the red ones, routes using published or random intermediate point may be selected.

The idea of FRA implementation in Europe appeared in 2008 and was introduced by Eurocontrol—Network Manager. Portugal was the first country in Europe to deploy this idea in its full version. Since then the introduction of FRA in Europe is a step-by-step process. Most states started with a limited implementation (e.g., during night hours) with an explicit view to gradually expand it. At the end of the year 2020, according to [31] most of the European countries have FRA effective 24 hours a day. At this time, however, FRA is still planned to be implemented in Great Britain, France, Spain, Czech Republic or Ukraine. To compare by the end of the year 2023 expected FRA implementation should cover almost entire Europe. Only FIR regions EGTT—FIR London in the UK and LFRR—FIR Brest in France remain in the plan zone.

It is also worth mentioning that according to [1] Free Route shall be provided and operated in the airspace for which the Member States are responsible at and above flight level 310 (FL310) in the ICAO EUR region from 1 January 2022 (as mentioned before). Although similar initiatives may be found in other parts of the world (such as North America or Australia), Europe is the first region in the world to have implemented a complete free route airspace approach. Its origins may be found in [32].

**Figure 3:**
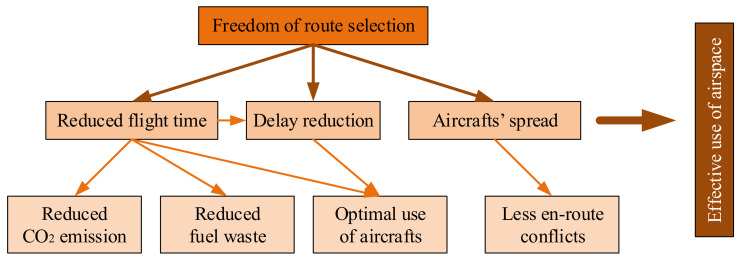
Benefits of FRA implementation and their relation [own work].

Implementation of a new functionality brings a number of benefits on one hand and a number of challenges on the other hand. The profits are (see Figure 3):

- freedom (even if gently limited in practice) of route selection,
- reduced flight time, as an effect of choosing the shortest routes possible,
- air quality consideration - reduced CO_2_ (and other gases) emission and fuel waste, as a consequence of reduced flight time,
- delay reduction,
- effective use of airspace, less conflicts, as an effect of aircrafts spread over bigger area.

**Figure 4:**
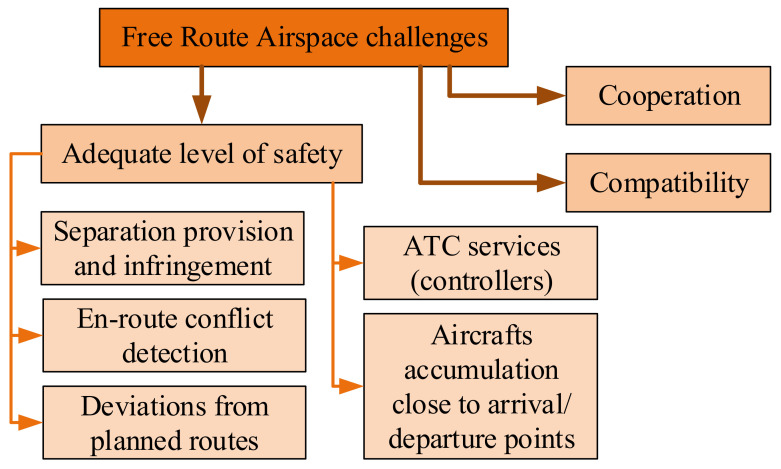
Challenges of FRA implementation [own work].

The challenges (Figure 4) on the other hand refer to issues related to:

- safety assurance—the main concern in air transport; the existing high safety level must not be deteriorated due to implementation of a new ATM functionality,
- separation provision and infringement, adequate use of collected data,
- en-route conflict detection and aircrafts accumulation close to FRA arrival and departure points (close to aerodromes areas),
- ATC (controllers) work,
- deviations from planned routes (due to weather, navigation fault, etc.),
- compatibility between ATC (Air Traffic Control) sectors and other ATM functionalities as well as cooperation with other neighboring countries as well as between FIRs as the bigger the airspace with free route selection the bigger the benefits of its implementation.

In further analysis Poland, and more precisely FIR Warszawa, was selected as a reference and natural analysis location. However, it must be noted that similar problem’s approach, hazard’s identification and analysis could be conducted for any another European country, as ATM system’s modifications arise from EU regulations and solutions implemented in the member countries are often the same. Especially in air transport, due to its transborder and international character, applied solution must be compatible. POLFRA (FRA in Poland) general rules and detailed boundaries together with the restrictions for free routing in available airspace can be found on [29] as well as in [2].

## 3. Failure Mode and Effects Analysis of POLFRA Implementation

Based on the two previous paragraphs, it can be said, that a new functionality has been added to the polish ATM. Does it influence ATM system and its safety? Being aware of the strict rules and procedures being a fact in air transport, it can be stated with full responsibility, that adequate safety analysis must have been carried out before implementation of this new solution. Is that enough for the entire free route idea functioning? Is it sufficient to check once before implementation? Does it comply with safety management standards recommending systematic, transparent and proactive approach to safety issues? Of course, not! Necessary analysis must be carried out not only before the implementation but also during new process’ functioning in a cyclic way. That is why, to ensure compliance with SMS requirements (especially proactivity and regularity) the following part of this article concentrates on the Failure Mode and Effects Analysis (FMEA) of free route airspace implementation in Poland (as an example location).

As mentioned in [2] this issue has already been addressed. Till now FRA rules and procedures were described and the selection of risk analysis method was justified. However, it is worth emphasizing that: FMEA analysis is a technique used to identify ways in which components, systems or processes can fail and not fulfil their tasks. The obtained results were presented in tabular form, identifying component’s name, potential incompatibilities, their possible effects and causes as well as recommended corrective actions. Nevertheless, the information presented therein were descriptive only. As a continuation, a similar table, however, expanded and complemented will be introduced as Table 5, as it is time to determine the three main *RPN* components rating scales for consequence of failure—*S*, probability of its occurrence—*P* and the ability to detect the problem—*W* then calculate the Risk Priority Number and show a quantitative result, indicating criticality of the identified potential failures. The descriptions in the table (especially for the possible effect) stay in direct relation with the challenges identified in paragraph 2.2, as they both concern safety, separation, ATC errors and route planning. Moreover, they are helpful for *P*, *S* and *W* assessment. Analysis is carried out from data/information point of view, as the entire ATM management is based on reliable, punctual and integral data exchange. Distinguished components are therefore arranged in three groups, distinguishing data/information preparation, it operational use and compatibility with other systems and countries (FIRs).

### 3.1. RPN Components Criteria Determination

Risk Priority Number (*RPN*) calculation is one of the quantitative measures, allowing classification of identified failures/incompatibilities according to their criticality to the system. In other words, its calculations allow arrangement of potential incompatibilities according to their significance to the entire system’s functioning at the same time pointing out which incompatibilities are to be taken care of in first place. Its three main components are:

- probability/risk of incompatibility occurrence—*P*,
- consequence/effect of failure—*S*,
- ability to detect the problem—*W*.

Rating scales of those three measures vary in different applications, according to literature review. In some FMEA analysis they accept values from 1 to 4 (based on [33]) or up to 5 (as in the risk tolerability matrix, from [34]). However, in most cases, as observed in [35,36,37] for example each of components is rated from 1 to 10, thereby affecting the *RPN* number to be assigned a value from 1 to 1000. Based on the analyzed international standards ([33,38]), literature ([36,37]) and various consultations with ATM system’s personnel in this considerations the following 10-value scales for parameters *P*, *S* and *W* were adopted—Table 1, Table 2 and Table 3.

### 3.2. RPN Determination and Interpretation

Once the components criteria are defined, adequate values to each of the potential failures/incompatibilities may be assigned and the results interpreted. Multiplication of three component values—*P*, *S* and *W*, each rated from 1 to 10, gives the *RPN* value within range 1 to 1000. The bigger the *RPN* number the higher the criticality of the incompatibility defined. Consequently, the corrective actions for failures with bigger *RPN* number should be introduced in the first line.

*RPN* interpretation was adopted in accordance with [37], which means that all system/process parts with *RPN* number equal or greater than 100 may be a major threat (see Table 4).

That is why in Table 5, showing aggregated results, potential incompatibilities with *RPN* value greater than 100 are highlighted with soft red color. They require action in first place. The rows marked with light green have *RPN* number minor to 100, so those ATM system phases do not cause a significant threat at the moment. No corrective actions are actually necessary, their monitoring is sufficient as prevention.

For the purposes of this analysis three independent experts were asked for opinion and assessment of described values *P*, *S* and *W* according to the interpretation given in Table 1, Table 2 and Table 3. Which means that if any of them assessed for example variable *S* to 10 (very dangerous), he accepted the interpretation/justification given in Table 2. The same procedure refers to all the three variables as well as to the each assessed incompatibility, no matter if it was evaluated to 1, 4 or 10.

## 4. Results and Discussion

The aggregated results of the FMEA analysis for POLFRA implementation are shown in Table 5.

Additionally, to provide an illustrative knowledge concerning detailed assessments of all three experts, selection of their opinions—limited to point a in each identified category—is shown in a brief form in Table 6.

To comment the provided Table 6, three selected lines (first lines 1a, 2a and 3a) are analyzed. In the first discussed case (1a first line) experts’ no 1 and 2 opinions were consistent. Expert no 3 assessed probability and detection one/two point more severely and possible effects less strictly. In effect, taking into account the slight discrepancy noticed in the aggregated table (Table 5) ‘Incorrect determination of entry, exit and intermediate points’ was assessed to the higher criteria given for *P* and *S* and a medium value of 3 for *W*.

In the second described case the experts are quite coherent, when assessing *P* and *W* values. Bigger discrepancies may be noticed in case of possible consequences *S* value. Expert no 1 gave the lightest rating—5, while Expert no 3 the most severe rating—8. In effect S value was finally assessed to 7 (in accordance with Expert’s no 2 opinion).

In the third analyzed case Experts’ no 2 and 3 opinions are compatible, while Expert no 1 presented more restrictive judgement especially in case of variables *P* and *W*. In effect those two variables must have been given higher values adequately 5 and 2.

To sum this discussion up it can be noted that in risk assessment small differences in experts’ opinion should not be surprising. The assessment is individual as each of the experts bases his opinion on his professional experience. That’s why the criteria assigned may vary. In effect the values of *RPN* number, presented in Table 5, as in each risk analysis may be subjective. If they were to be used operationally, it would be advised to increase the number of independent experts involved in the assessment. However, in this first approach, showing the procedure and identifying the components rating scales, it was decided that involvement of three experts will allow drawing reliable observations. And these are:

- the values obtained in the analysis are generally not big, the maximum value that appeared is *RPN* = 135,
- from fifteen (15) potential failures/incompatibilities identified five (5) exceeded the *RPN* limit value equal to 100 (Table 7), and out of those five (5) three (3) values belong to process stage number 2—operational use of published aeronautical data and information,
- at the 2nd stage—operational use of published aeronautical data and information, high rates (7–10) of variable *S*, responsible for consequence of incompatibilities appearance were noted, if not detected leading to air accidents and aircrafts’ damage; the situation is mitigated thanks to assessed high ability to detect the failure, thanks to which the *RPN* value does not increase significantly,
- in the 3rd group—compatibility, none of the values obtained exceeds 100, this time thanks to lower consequence rates at similar level of the other two components,
- the highest *RPN* values = 135 and 120 are obtained for ‘Aircrafts separation minima infringement’, for which the effect was rated as nine (9) with the probability set at the level of five (5) as well as for ‘Exit from POLFRA area into the airport’s CTR (Controlled Area) through other point then defined in arrival connecting point’, for which, due to high aircrafts’ density, the consequences were rated the maximum value ten (10), the probability is equal to four (4) and detection was assessed to be three (3),
- what also worries is the possibility of non-compliance appearance in AIP Polska publication (the remaining two cases of *RPN* value over 100), as operational use of incorrect data may be fraught with consequence and AIP Polska publication is a basis for safe preparation and conduct of flights.

## 5. Conclusions

ATM modernization goes hand in hand with the development of air transport. New functionalities are being added to enable a more efficient use of airspace, to improve runway safety and throughput, ensure benefits in terms of fuel consumption and delay reduction as well as airport capacity. Whether the function is new or not its deployment and operational use must not influence the high level of safety worked out and expected in air traffic and transport. Moreover, safety management must comply with the rules of being proactive, systematic and transparent. In this paper Flexible Airspace Management and Free Route function was addressed as the possibility to freely plan a route between a defined entry point and a defined exit point appeared in Poland only in February 2019. Conducted risk analysis based on FMEA methodology with Risk Priority Number calculation allowed lining up of the identified potential incompatibilities according to their criticality to the system. Thanks to the three main *RPN* components rating scales determination, the FMEA risk analysis of FRA implementation gives back a quantitative result. At the same time, with all this done the main article’s research goal, presented in the introduction, is achieved. The case study is carried out in Poland as FRA deployment location, however, as explained in the text, similar results would be expected in any other European country.

The results are widely discussed in paragraph four. To conclude it can be said that the question posed was whether the free route implementation (in Poland) influences air transport safety? In authors opinion the answer is positive—yes, it does. Nevertheless, this influence does not mean negative impact. Generally, it could be said that each modification in a management system, related to safety, influence the safety itself. However, the changes and improvements are an indispensable part in technical systems. Moreover, what can also be noted in managing safety is the contemporaneous change of the safety context, from its “hard” formula—technical means, resources, personnel, etc., to a “soft” one—immaterial assets including information, hazards. Not high maximum *RPN* value obtained in the conducted analysis is a positive piece of information. This suggests that the deployment was prepared carefully. Although, it must still be monitored. What else can be done, based on the potential incompatibilities causes identified? A complex, systematic approach to aeronautical data and information management is required. Dedicated solutions should be applied on all stages of the aeronautical data and information chain. Not only in its last phase—operational use but much earlier, starting from data request and origination in order to request and create correct and necessary data, which will be processed and used later on.

What is also worth summarizing is that values of incompatibilities appearance and their detection as well as consequence severity (*P*, *W* and *S*) in most cases are dependent on the human factor. As demonstrated repeatedly the human error is the reason of most of the incidents/accidents not only in the air but generally in all transport types. This remains in tune with the *P*, *S* and *W* values shown in Table 5. The probability differs around a medium value (5–6), severity is high (7–10), while detection is a means to restrain the unfavorable impact. The consequences—*S* in air transport—are usually significant. Luckily air traffic accidents are rare, thanks to the proactive safety approach as well as error appearance mitigation, thanks to their correct detection, noticeable in *W* column of Table 5.

In the future research the risk analysis may also be carried out for other ATM functionalities listed in the Pilot Common Project. The next one in terms of authors’ interest is functionality No 5—Initial System Wide Information Management, as nowadays information exchange is a basis for each activity, also for a safe preparation on conduct of flights. And taking into account the mentioned growth in operations’ and passengers’ numbers, which have direct impact on information to be processed, the issue of aeronautical data and information quality assurance in the aspects of operational risk and safety is still actual and important.

## Figures and Tables

**Table 1:** Criteria for probability/risk of incompatibilities’ appearance assessment, based on [33,36,37].

Incompatibility/Error in POLFRA Implementation	Variable *P* Value	Criterion Description
Improbable	1	Incompatibilities’ appearance is improbable. Process with very high quality.
Rare	2–3	Incompatibilities’ appearance is very rare but should be noted even if does not pose a significant threat.
Reasonable	4–6	Incompatibility occurs occasionally but you have to reckon with it.
Often	7–8	Incompatibility occurs often and is a significant threat. Special action required.
Very often	9–10	Incompatibility (almost) cannot be avoided. Process with low quality.

**Table 2:** Criteria for consequences/effects of incompatibilities’ appearance, own study based on [4,33,36].

Severity of Consequences/Effects	Variable *S* Value	Criterion Description
None	1	Air incident: aircraft’s airworthiness maintained - uninterrupted air operation.
Meaningless	2	Air incident: aircraft’s airworthiness maintained after confirmation - disturbed air operation.
Very small	3	Air incident: aircraft’s airworthiness lost—interrupted air operation, aircraft’s repair.
Small	4	Air incident: aircraft’s airworthiness lost—interrupted air operation, aircraft’s damage and repair.
Moderate	5	Serious air incident due to a collision with a bird or vehicle in the ground traffic area—aircraft’s damage and repair.
Significant	6	Serious incident resulting from a collision with a bird or vehicle in the ground traffic area or a collision with an obstacle or an element of aerodrome’s infrastructure—aircraft’s damage and repair.
Big	7	Serious incident resulting from a serious collision or involving two (or more) aircrafts.
Very big	8	Air accident due to a collision with a bird or vehicle in the ground traffic area—aircraft’s damage and repair.
Dangerous	9	Air accident resulting from a collision with a bird or vehicle in the ground traffic area or a collision with an obstacle or an element of aerodrome’s infrastructure—aircraft’s damage.
Very dangerous	10	Air accident resulting from a serious collision or involving two (or more) aircrafts - aircraft’s damage.

**Table 3:** Criteria for incompatibilities’ detection, own study based on [33,36].

Incompatibilities’ Detection	Variable *W* Value	Criterion Description
Almost certain	1	Process control will almost certainly detect the potential incompatibility/failure. There are clear symptoms of error’s appearance.
Very high	2	There is a very good chance that process control will detect the potential incompatibility/failure. There are clear symptoms of error’s appearance.
High	3	There is a good chance that process control will detect the potential incompatibility/failure. There are noticeable symptoms of error’s appearance.
Slightly high	4	There is a slightly good chance that process control will detect the potential incompatibility/failure. There are noticeable symptoms of error’s appearance.
Moderate	5	There is a moderate chance that process control will detect the potential incompatibility/failure. Symptoms of error’s appearance may be noted.
Low	6	There is a small chance that process control will detect the potential incompatibility/failure. Symptoms of error’s appearance may be noted.
Very low	7	There is a very small chance that process control will detect the potential incompatibility/failure. Symptoms of error’s appearance are imperceptible.
Unlikely	8	Detection of the potential incompatibility/failure is unlikely. Symptoms of error’s appearance are imperceptible.
Very unlikely	9	Detection of the potential incompatibility/failure is very unlikely. Symptoms of error’s appearance are imperceptible.
Absolutely unlikely	10	Process control will/may not detect the potential incompatibility/failure or there is lack of process verification means. No symptoms of error appearance.

**Table 4:** Criteria for probability/risk of incompatibilities’ appearance assessment, based on [33,36,37].

*RPN* Value	Criterion Description
1–99	Specified incompatibility does not cause a significant threat for (POL)FRA deployment and application
100–1000	Incompatibility is/may be a major threat for (POL)FRA deployment and application

**Table 5:** FMEA analysis for POLFRA implementation, own work.

No	Component’s Name	Potential Incompatibility	Possible Effect	Potential Cause	Corrective Actions	*P*	*S*	*W*	*RPN*
**1**	**Aeronautical data and information preparation**								
1a	Determination of entry, exit and intermediate points	Incorrect determination of entry, exit and intermediate points	Creation of incorrect data and charts; serious incident involving two (or more) aircrafts	Lack of verification procedures; lack of people competence; misunderstanding; human error	Implementation of verification procedures; careful study of the documentation; designation of the competent person	3	7	3	63
		Incompatibility in AIP Polska publication	Lack of information publication in a correct form, place and time; operational use of incorrect data; aeronautical accident involving two (or more) aircrafts; aircraft’s destruction	Incompatibilities in the aeronautical data and information chain; no stage verification; ignorance of rules and requirements; human error	Data and information verification; implementation of verification procedures on subsequent stages	4	10	3	120
1b	Determination of departure/arrival connecting points	Incorrect determination of departure/arrival connecting points	Creation of incorrect data and charts; serious incident involving two (or more) aircrafts	Lack of verification procedures; lack of people competence; misunderstanding; human error; ignorance of TMA boundaries	Implementation of verification procedures; careful study of the documentation; designation of the competent person	3	7	3	63
		Incompatibility in AIP Polska publication	Lack of information publication in a correct form, place and time; operational use of incorrect data; aeronautical accident involving two (or more) aircrafts; aircraft’s destruction	Incompatibilities in the aeronautical data and information chain; no stage verification; ignorance of rules and requirements; human error	Data and information verification; implementation of verification procedures on subsequent stages	4	9	3	108
1c	Publication of information in different forms	Inconsistency between published written information and published charts	Operational use of incorrect data, serious incident involving two (or more) aircrafts	Lack of verification procedures; lack of people competence; human error	Data and information verification; designation of the competent person	4	8	2	64
1d	DCT (Direct Routing) determination	Incompatibility in DCTs publication	Incorrect route planning through restricted areas	Lack of verification procedures; lack of people competence; human error	Data and information verification; designation of the competent person	5	10	1	50
**2**	**Operational use of published aeronautical data and information**								
2a	Route planning	Route planning through areas where POLFRA is not applicable: DA, TMA, CTR	Serious incident involving two (or more) aircrafts; aircraft’s destruction	Incompatibilities on the 1st stage - aeronautical data and information preparation; ignorance of the current situation; human error	Implementation of the verification procedures on the 1st stage; detection of conflicts in planned routes	4	7	1	28
		Entry into POLFRA area from airports located in FIR Warszawa from other point then defined in departure connecting point	Traffic congestion around defined connecting points; aircrafts separation minima infringement; serious incident involving two (or more) aircrafts	Incompatibilities on stage 1b; human error	Implementation of the verification procedures on the 1b stage; good knowledge of published points; correct route planning	4	9	3	108
		Exit from POLFRA area into the airport’s CTR through other point then defined in arrival connecting point	Safety risk; aeronautical accident involving two (or more) aircrafts; aircraft’s destruction	Incompatibilities on stage 1b; human error	Implementation of the verification procedures on the 1b stage; good knowledge of published points; correct route planning	4	10	3	120
2b	Traffic management	Traffic congestion around defined entry/exit points	Serious incident involving two (or more) aircrafts; negative effects for the environment; additional use of fuel	Incorrect determination of entry, exit and intermediate points; errors on stage 1a; incorrect route planning	Implementation of the verification procedures on the 1a stage; correct route planning; correct traffic management	6	7	1	42
		Aircrafts separation minima infringement	Serious incident involving two (or more) aircrafts	Traffic congestion around defined entry/exit points; incorrect route planning; ATC error; human error	Correct traffic management	5	9	3	135
		Conflicts in planned routes	Urgent need to re-plan the route; delays, dissatisfaction; if not detected - aeronautical accident involving two (or more) aircrafts; aircraft’s destruction	Incompatibilities on the 1st stage - aeronautical data and information preparation; lack of conflict detection tool	Implementation of conflict detection tool	4	10	2	80
**3**	**Compatibility**								
3a	Cross-border arrangements	Implementation delays	Incomplete application of the expected effects; dissatisfaction	Indolence; lack of competences	Proactive approach and active operation	5	2	2	20
		Lack of compatibility of POLFRA significant points and DCTs located on FIR Warszawa boundaries with other countries arrangements (Baltic FAB, Germany, Ukraine, Scandinavia)	Impossible deployment of FRA on international level; incomplete application of the expected effects	Lack of necessary arrangements; indolence	Active operation	5	3	5	75
3b	Interdependencies and compatibility with other ATM functions/systems	Lack of compatibility with other ATM functions and systems (such as SWIM, ATFCM, etc.)	Impossible full deployment of FRA on national and international level	Lack of necessary arrangements; indolence	Active operation; need for synchronization; implementation of other required functionalities	4	2	5	40

**Table 6:** Selected assessments of three experts in a brief form, own work.

No	Component’s Name	Potential Incompatibility	Expert no 1			Expert no 2			Expert no 3		
			*P*	*S*	*W*	*P*	*S*	*W*	*P*	*S*	*W*
1a	Determination of entry, exit and intermediate points	Incorrect determination of entry, exit and intermediate points	2	7	2	2	7	2	3	6	4
		Incompatibility in AIP Polska publication	3	8	2	5	10	3	3	10	3
2a	Route planning	Route planning through areas where POLFRA is not applicable: DA, TMA, CTR	4	5	2	4	7	1	2	8	1
		Entry into POLFRA area from airports located in FIR Warszawa from other point then defined in departure connecting point	4	7	3	4	10	2	4	9	3
		Exit from POLFRA area into the airport’s CTR through other point then defined in arrival connecting point	4	8	3	4	10	3	4	10	2
3a	Cross-border arrangements	Implementation delays	6	1	4	4	1	1	4	2	1
		Lack of compatibility of POLFRA significant points and DCTs located on FIR Warszawa boundaries with other countries arrangements (Baltic FAB, Germany, Ukraine, Scandinavia)	5	3	4	4	3	4	4	2	6

**Table 7:** Summary of FMEA analysis for POLFRA implementation, own work.

*RPN* Value		Number of Results Obtained	Number of Results Obtained in %
1–99		10	66.66 %
100–1000		5	33.33 %
	Total:	15	100 %

## Data Availability

Not applicable.
